# Supplementary figures and images for: Prediction of metastatic prostate cancer by prostate-specific antigen in combination with T stage and Gleason Grade: Nationwide, population-based register study
Source: PLoS One. 2020 Jan 29;15(1):e0228447. doi: 10.1371/journal.pone.0228447 (PMC6988964; doi:10.1371/journal.pone.0228447)

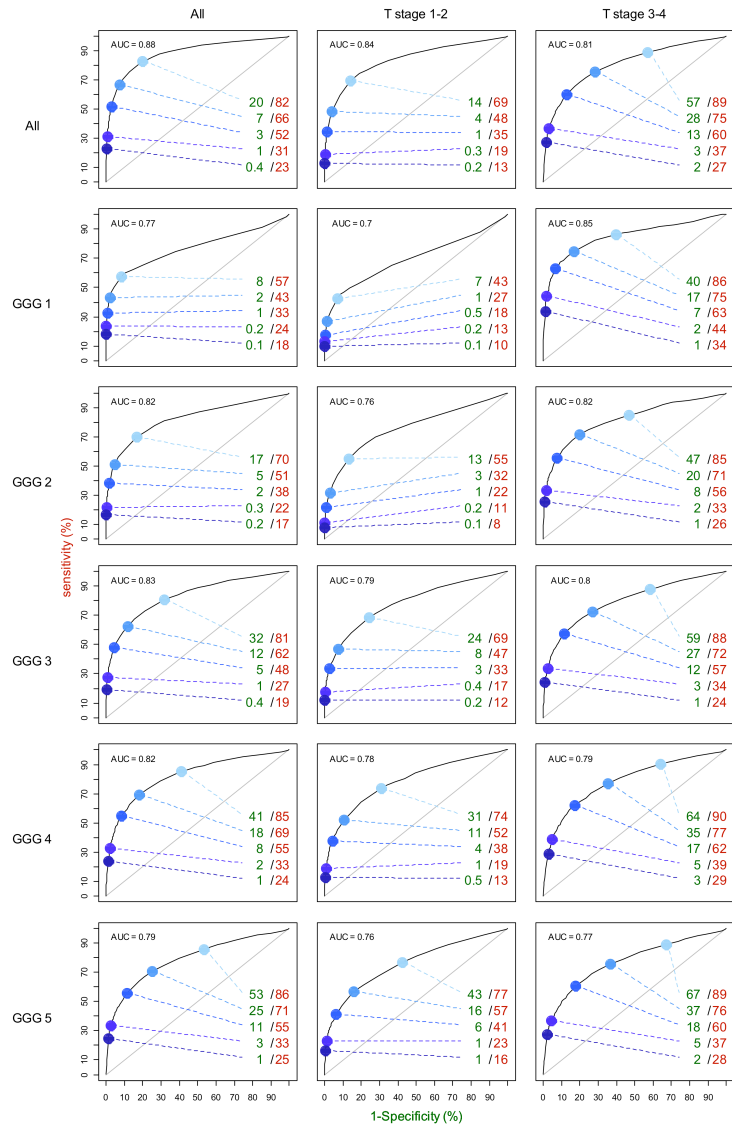

■ PSA = 20 ■ PSA = 50 ■ PSA = 100 ■ PSA = 300 ■ PSA = 500

Supplement: S1 Fig — (PDF) [file pone.0228447.s001.pdf]
