# Supplementary material for: Prediction of metastatic prostate cancer by prostate-specific antigen in combination with T stage and Gleason Grade: Nationwide, population-based register study
Source: PLoS One. 2020 Jan 29;15(1):e0228447. doi: 10.1371/journal.pone.0228447 (PMC6988964; doi:10.1371/journal.pone.0228447)
Supplement: S1 Table — (DOCX) [file pone.0228447.s002.docx]

| **Supplemental Table 1** Total number of men in subgroups defined by prostate-specific antigen (PSA), Gleason grade groups (GGG) and T stage and number of men with metastatic prostate cancer (percentage of men with metastases with metastatic prostate cancer in the subgroup) | | | | | | | |
| --- | --- | --- | --- | --- | --- | --- | --- |
| T1-4 |  |  |  |  |  |  |  |
|  | Any PSA | PSA 0-20 | PSA 20-50 | PSA 50-100 | PSA 100-300 | PSA 300-500 | PSA 500+ |
| GGG 1-5 | 101729 | 74668 | 12625 | 5587 | 4488 | 1308 | 3054 |
| Metastases (%) | 12157 (12) | 2228 (3) | 1904 (15) | 1779 (32) | 2526 (56) | 1012 (77) | 2708 (89) |
| GGG 1 | 39770 | 36494 | 2296 | 543 | 224 | 66 | 146 |
| Metastases (%) | 455 (1) | 200 (1) | 60 (3) | 46 (8) | 41 (19) | 29 (47) | 78 (58) |
| GGG 2 | 24220 | 19909 | 2727 | 857 | 460 | 81 | 186 |
| Metastases (%) | 888 (4) | 278 (1) | 157 (6) | 115 (13) | 147 (32) | 46 (57) | 145 (79) |
| GGG 3 | 14211 | 8873 | 2672 | 1153 | 851 | 221 | 441 |
| Metastases (%) | 2044 (14) | 414 (5) | 368 (14) | 289 (25) | 419 (49) | 166 (75) | 388 (88) |
| GGG 4 | 11350 | 5340 | 2286 | 1282 | 1201 | 359 | 882 |
| Metastases (%) | 3322 (29) | 515 (10) | 515 (23) | 489 (38) | 732 (61) | 287 (80) | 784 (89) |
| GGG 5 | 12178 | 4052 | 2644 | 1751 | 1752 | 581 | 1399 |
| Metastases (%) | 5450 (45) | 821 (20) | 805 (30) | 840 (48) | 1187 (68) | 483 (83) | 1313 (94) |
| T1-2 |  |  |  |  |  |  |  |
|  | Any PSA | PSA 0-20 | PSA 20-50 | PSA 50-100 | PSA 100-300 | PSA 300-500 | PSA 500+ |
| GGG 1-5 | 81366 | 68273 | 8155 | 2475 | 1474 | 342 | 647 |
| Metastases (%) | 3972 (5) | 1266 (2) | 804 (10) | 534 (22) | 629 (43) | 234 (69) | 505 (78) |
| GGG 1 | 38190 | 35595 | 1961 | 385 | 133 | 36 | 79 |
| Metastases (%) | 302 (1) | 179 (1) | 42 (2) | 28 (7) | 13 (10) | 11 (33) | 29 (44) |
| GGG 2 | 21262 | 18448 | 2029 | 484 | 215 | 27 | 59 |
| Metastases (%) | 451 (2) | 209 (1) | 98 (5) | 46 (10) | 48 (23) | 13 (51) | 35 (63) |
| GGG 3 | 10179 | 7508 | 1636 | 544 | 316 | 63 | 112 |
| Metastases (%) | 778 (8) | 255 (3) | 168 (10) | 98 (18) | 122 (39) | 45 (71) | 90 (80) |
| GGG 4 | 6577 | 4182 | 1299 | 485 | 371 | 87 | 153 |
| Metastases (%) | 1017 (15) | 277 (7) | 217 (17) | 143 (30) | 189 (51) | 65 (74) | 126 (83) |
| GGG 5 | 5159 | 2541 | 1230 | 577 | 440 | 128 | 244 |
| Metastases (%) | 1424 (28) | 346 (14) | 279 (23) | 218 (38) | 256 (58) | 101 (79) | 224 (92) |
| T3-4 |  |  |  |  |  |  |  |
|  | Any PSA | PSA 0-20 | PSA 20-50 | PSA 50-100 | PSA 100-300 | PSA 300-500 | PSA 500+ |
| GGG 1-5 | 20363 | 6394 | 4470 | 3112 | 3013 | 966 | 2407 |
| Metastases (%) | 8187 (40) | 963 (15) | 1101 (25) | 1245 (40) | 1897 (63) | 778 (81) | 2203 (92) |
| GGG 1 | 1580 | 900 | 335 | 157 | 92 | 30 | 67 |
| Metastases (%) | 153 (10) | 21 (2) | 18 (5) | 18 (11) | 28 (31) | 18 (62) | 49 (74) |
| GGG 2 | 2959 | 1461 | 698 | 374 | 245 | 54 | 128 |
| Metastases (%) | 437 (15) | 69 (5) | 59 (8) | 68 (18) | 99 (40) | 33 (61) | 110 (86) |
| GGG 3 | 4032 | 1364 | 1036 | 609 | 535 | 158 | 329 |
| Metastases (%) | 1266 (31) | 159 (12) | 200 (19) | 191 (31) | 296 (55) | 122 (77) | 298 (91) |
| GGG 4 | 4774 | 1158 | 988 | 797 | 829 | 272 | 729 |
| Metastases (%) | 2305 (48) | 238 (21) | 298 (30) | 346 (43) | 543 (65) | 222 (82) | 658 (90) |
| GGG 5 | 7018 | 1511 | 1414 | 1175 | 1312 | 453 | 1154 |
| Metastases (%) | 4025 (57) | 475 (31) | 526 (37) | 622 (53) | 931 (71) | 383 (85) | 1088 (94) |
