# Supplementary material for: Prediction of metastatic prostate cancer by prostate-specific antigen in combination with T stage and Gleason Grade: Nationwide, population-based register study
Source: PLoS One. 2020 Jan 29;15(1):e0228447. doi: 10.1371/journal.pone.0228447 (PMC6988964; doi:10.1371/journal.pone.0228447)
Supplement: S2 Table — (DOCX) [file pone.0228447.s003.docx]

| **Supplemental Table 2** Sensitivity, 1-specificity, positive predictive value (PPV), negative predictive value (NPV), Positive likelihood ratio (LRH+), negative likelihood ratio (LRH-) for predicting metastases in men with T1-2 prostate cancer | | | | | | | | | | | | | |  |
| --- | --- | --- | --- | --- | --- | --- | --- | --- | --- | --- | --- | --- | --- | --- |
| PSA | GGG | Sensitivity (95% CI) | | 1-specificity (95% CI) | | PPV (95% CI) | | NPV (95% CI) | | LRH+ (95% CI) | | LHR- (95% CI) | | |
| 20 | GGG 1 | 69 | (67 - 71) | 14 | (13 - 15) | 20 | (19 - 21) | 98 | (98 - 98) | 5 | (4 - 5) | 0.36 | (0.34 - 0.38) | |
| 20 | GGG 2 | 43 | (33 - 52) | 7 | (4 - 11) | 5 | (4 - 6) | 100 | (99 - 100) | 6 | (4 - 9) | 0.62 | (0.53 - 0.72) | |
| 20 | GGG 3 | 55 | (47 - 62) | 13 | (10 - 17) | 8 | (7 - 10) | 99 | (99 - 99) | 4 | (3 - 5) | 0.52 | (0.45 - 0.6) | |
| 20 | GGG 4 | 69 | (63 - 73) | 24 | (21 - 28) | 19 | (17 - 21) | 97 | (96 - 97) | 3 | (2 - 3) | 0.41 | (0.36 - 0.48) | |
| 20 | GGG 5 | 74 | (69 - 77) | 31 | (28 - 34) | 30 | (28 - 33) | 93 | (92 - 94) | 2 | (2 - 3) | 0.38 | (0.33 - 0.44) | |
| 50 | GGG 1 | 48 | (46 - 50) | 4 | (3 - 5) | 38 | (36 - 40) | 97 | (97 - 97) | 12 | (10 - 14) | 0.54 | (0.52 - 0.56) | |
| 50 | GGG 2 | 27 | (17 - 38) | 1 | (0.4 - 4) | 13 | (9 - 16) | 99 | (99 - 100) | 18 | (7 - 48) | 0.74 | (0.66 - 0.83) | |
| 50 | GGG 3 | 32 | (25 - 40) | 3 | (2 - 6) | 18 | (15 - 21) | 99 | (98 - 99) | 10 | (6 - 17) | 0.7 | (0.64 - 0.77) | |
| 50 | GGG 4 | 47 | (41 - 52) | 8 | (6 - 10) | 34 | (30 - 37) | 95 | (95 - 96) | 6 | (5 - 8) | 0.58 | (0.53 - 0.63) | |
| 50 | GGG 5 | 52 | (47 - 56) | 11 | (9 - 13) | 47 | (44 - 50) | 91 | (90 - 92) | 5 | (4 - 6) | 0.54 | (0.49 - 0.59) | |
| 100 | GGG 1 | 57 | (53 - 60) | 16 | (14 - 19) | 57 | (54 - 60) | 84 | (82 - 85) | 4 | (3 - 4) | 0.52 | (0.48 - 0.56) | |
| 100 | GGG 2 | 35 | (33 - 37) | 1 | (1 - 2) | 55 | (49 - 61) | 97 | (97 - 97) | 24 | (17 - 34) | 0.66 | (0.64 - 0.68) | |
| 100 | GGG 3 | 18 | (9 - 28) | 0.5 | (0.0 - 3) | 22 | (12 - 34) | 99 | (99 - 99) | 35 | (6 - 211) | 0.83 | (0.75 - 0.91) | |
| 100 | GGG 4 | 22 | (16 - 28) | 1 | (0.3 - 3) | 32 | (25 - 40) | 98 | (98 - 99) | 22 | (8 - 58) | 0.79 | (0.74 - 0.85) | |
| 100 | GGG 5 | 33 | (28 - 39) | 3 | (1 - 4) | 52 | (46 - 57) | 95 | (94 - 95) | 13 | (8 - 21) | 0.68 | (0.64 - 0.73) | |
| 200 | GGG 1 | 38 | (33 - 42) | 4 | (3 - 6) | 62 | (57 - 66) | 89 | (88 - 90) | 9 | (6 - 12) | 0.65 | (0.61 - 0.69) | |
| 200 | GGG 2 | 41 | (37 - 44) | 6 | (5 - 8) | 71 | (67 - 75) | 81 | (79 - 82) | 6 | (5 - 8) | 0.63 | (0.6 - 0.67) | |
| 200 | GGG 3 | 24 | (23 - 26) | 0.5 | (0.3 - 1) | 70 | (59 - 78) | 96 | (96 - 96) | 45 | (24 - 83) | 0.76 | (0.74 - 0.78) | |
| 200 | GGG 4 | 15 | (7 - 26) | 0.2 | (0.0 - 3) | 35 | (14 - 57) | 99 | (99 - 99) | 66 | (4 - 1022) | 0.85 | (0.77 - 0.93) | |
| 200 | GGG 5 | 16 | (11 - 22) | 0.3 | (0.0 - 2) | 50 | (33 - 65) | 98 | (98 - 98) | 47 | (8 - 263) | 0.84 | (0.8 - 0.89) | |
| 300 | GGG 1 | 23 | (18 - 28) | 0.8 | (0.3 - 2) | 69 | (61 - 77) | 94 | (93 - 95) | 27 | (12 - 63) | 0.78 | (0.74 - 0.82) | |
| 300 | GGG 2 | 25 | (21 - 30) | 2 | (0.9 - 3) | 73 | (67 - 79) | 88 | (87 - 89) | 15 | (9 - 25) | 0.76 | (0.72 - 0.8) | |
| 300 | GGG 3 | 29 | (26 - 33) | 2 | (1 - 3) | 84 | (79 - 88) | 78 | (77 - 80) | 14 | (9 - 21) | 0.72 | (0.69 - 0.76) | |
| 300 | GGG 4 | 19 | (17 - 20) | 0.3 | (0.1 - 0.7) | 75 | (63 - 84) | 96 | (96 - 96) | 59 | (27 - 130) | 0.81 | (0.8 - 0.83) | |
| 300 | GGG 5 | 13 | (6 - 23) | 0.2 | (0.0 - 3) | 39 | (15 - 63) | 99 | (99 - 99) | 79 | (3 - 1992) | 0.87 | (0.8 - 0.94) | |
| 400 | GGG 1 | 11 | (7 - 16) | 0.2 | (0.0 - 2) | 59 | (33 - 78) | 98 | (98 - 98) | 66 | (5 - 840) | 0.89 | (0.85 - 0.93) | |
| 400 | GGG 2 | 17 | (14 - 22) | 0.4 | (0.1 - 1) | 77 | (66 - 85) | 94 | (93 - 94) | 40 | (13 - 129) | 0.83 | (0.8 - 0.87) | |
| 400 | GGG 3 | 19 | (15 - 23) | 0.9 | (0.4 - 2) | 80 | (72 - 85) | 87 | (86 - 88) | 21 | (11 - 43) | 0.82 | (0.78 - 0.85) | |
| 400 | GGG 4 | 23 | (20 - 26) | 1 | (0.7 - 2) | 87 | (82 - 91) | 77 | (76 - 79) | 18 | (11 - 30) | 0.78 | (0.75 - 0.81) | |
| 400 | GGG 5 | 16 | (14 - 17) | 0.2 | (0.1 - 0.6) | 78 | (63 - 88) | 96 | (96 - 96) | 71 | (26 - 190) | 0.85 | (0.83 - 0.86) | |
| 500 | GGG 1 | 12 | (6 - 22) | 0.1 | (0.0 - 3) | 45 | (14 - 72) | 99 | (99 - 99) | 99 | (2 - 4627) | 0.88 | (0.81 - 0.95) | |
| 500 | GGG 2 | 9 | (5 - 14) | 0.1 | (0.0 - 2) | 66 | (28 - 87) | 98 | (98 - 98) | 88 | (3 - 2642) | 0.91 | (0.87 - 0.95) | |
| 500 | GGG 3 | 14 | (11 - 18) | 0.3 | (0.1 - 1) | 79 | (66 - 88) | 93 | (93 - 94) | 46 | (12 - 183) | 0.86 | (0.83 - 0.89) | |
| 500 | GGG 4 | 15 | (12 - 19) | 0.6 | (0.2 - 1) | 82 | (74 - 88) | 87 | (85 - 88) | 25 | (11 - 59) | 0.85 | (0.82 - 0.88) | |
| 500 | GGG 5 | 19 | (16 - 22) | 0.7 | (0.3 - 2) | 91 | (86 - 94) | 76 | (75 - 78) | 26 | (13 - 50) | 0.81 | (0.79 - 0.84) | |
| 1000 | GGG 1 | 13 | (12 - 15) | 0.2 | (0.0 - 0.6) | 80 | (62 - 90) | 96 | (96 - 96) | 75 | (25 - 226) | 0.87 | (0.86 - 0.88) | |
| 1000 | GGG 2 | 10 | (4 - 18) | 0.1 | (0.0 - 3) | 45 | (12 - 74) | 99 | (99 - 99) | 100 | (1 - 7842) | 0.9 | (0.84 - 0.96) | |
| 1000 | GGG 3 | 8 | (5 - 13) | 0.1 | (0.0 - 2) | 65 | (29 - 86) | 98 | (98 - 98) | 83 | (3 - 2684) | 0.92 | (0.89 - 0.96) | |
| 1000 | GGG 4 | 12 | (9 - 15) | 0.2 | (0.0 - 1) | 81 | (65 - 91) | 93 | (92 - 94) | 52 | (10 - 273) | 0.88 | (0.85 - 0.91) | |
| 1000 | GGG 5 | 13 | (10 - 17) | 0.5 | (0.1 - 1) | 83 | (74 - 90) | 86 | (85 - 87) | 27 | (10 - 73) | 0.88 | (0.85 - 0.91) | |
| **Abbreviation** PSA prostate-specific antigen; GGG Gleason Grade Group; CI confidence interval | | | | | | | | | | | | | |  |
