# Supplementary material for: Prediction of metastatic prostate cancer by prostate-specific antigen in combination with T stage and Gleason Grade: Nationwide, population-based register study
Source: PLoS One. 2020 Jan 29;15(1):e0228447. doi: 10.1371/journal.pone.0228447 (PMC6988964; doi:10.1371/journal.pone.0228447)
Supplement: S3 Table — (DOCX) [file pone.0228447.s004.docx]

| **Supplemental Table 3** Sensitivity, 1-specificity, positive predictive value (PPV), negative predictive value (NPV), Positive likelihood ratio (LRH+), negative likelihood ratio (LRH-) for predicting metastases in men with T3-4 prostate cancer | | | | | | | | | | | | | |  |
| --- | --- | --- | --- | --- | --- | --- | --- | --- | --- | --- | --- | --- | --- | --- |
| PSA | GGG | Sensitivity (95% CI) | | 1-specificity (95% CI) | | PPV (95% CI) | | NPV (95% CI) | | LRH+ (95% CI) | | LHR- (95% CI) | | |
| 20 | GGG 1 | 89 | (88 - 90) | 57 | (56 - 58) | 51 | (50 - 52) | 85 | (84 - 86) | 2 | (2 - 2) | 0.26 | (0.24 - 0.28) | |
| 20 | GGG 2 | 86 | (75 - 93) | 40 | (30 - 49) | 19 | (15 - 23) | 98 | (96 - 99) | 2 | (2 - 3) | 0.22 | (0.13 - 0.39) | |
| 20 | GGG 3 | 85 | (79 - 89) | 47 | (41 - 52) | 24 | (21 - 27) | 95 | (94 - 97) | 2 | (2 - 2) | 0.28 | (0.21 - 0.38) | |
| 20 | GGG 4 | 88 | (85 - 90) | 59 | (55 - 62) | 41 | (38 - 43) | 88 | (85 - 90) | 2 | (1 - 2) | 0.29 | (0.24 - 0.36) | |
| 20 | GGG 5 | 90 | (89 - 92) | 64 | (61 - 67) | 57 | (55 - 59) | 80 | (77 - 83) | 1 | (1 - 1) | 0.27 | (0.22 - 0.32) | |
| 50 | GGG 1 | 89 | (87 - 90) | 67 | (65 - 69) | 64 | (62 - 66) | 69 | (65 - 72) | 1 | (1 - 1) | 0.34 | (0.3 - 0.39) | |
| 50 | GGG 2 | 75 | (74 - 77) | 28 | (27 - 30) | 64 | (63 - 65) | 81 | (80 - 82) | 3 | (3 - 3) | 0.35 | (0.33 - 0.36) | |
| 50 | GGG 3 | 75 | (62 - 84) | 17 | (10 - 25) | 32 | (25 - 40) | 97 | (95 - 98) | 4 | (3 - 7) | 0.3 | (0.21 - 0.44) | |
| 50 | GGG 4 | 71 | (65 - 77) | 20 | (16 - 24) | 39 | (34 - 43) | 94 | (93 - 95) | 4 | (3 - 4) | 0.36 | (0.3 - 0.43) | |
| 50 | GGG 5 | 72 | (68 - 76) | 27 | (24 - 30) | 55 | (52 - 58) | 85 | (83 - 87) | 3 | (2 - 3) | 0.38 | (0.33 - 0.43) | |
| 100 | GGG 1 | 77 | (75 - 79) | 35 | (33 - 38) | 67 | (65 - 69) | 75 | (73 - 77) | 2 | (2 - 2) | 0.35 | (0.32 - 0.39) | |
| 100 | GGG 2 | 76 | (74 - 78) | 37 | (34 - 39) | 74 | (72 - 75) | 66 | (64 - 68) | 2 | (2 - 2) | 0.38 | (0.35 - 0.42) | |
| 100 | GGG 3 | 60 | (59 - 62) | 13 | (12 - 14) | 76 | (74 - 77) | 77 | (76 - 77) | 5 | (4 - 5) | 0.46 | (0.44 - 0.47) | |
| 100 | GGG 4 | 63 | (50 - 73) | 7 | (3 - 13) | 50 | (39 - 60) | 96 | (94 - 97) | 9 | (5 - 18) | 0.4 | (0.3 - 0.52) | |
| 100 | GGG 5 | 56 | (48 - 62) | 8 | (5 - 11) | 56 | (50 - 62) | 92 | (91 - 94) | 7 | (5 - 10) | 0.48 | (0.42 - 0.55) | |
| 200 | GGG 1 | 57 | (53 - 61) | 12 | (10 - 14) | 69 | (66 - 73) | 82 | (80 - 84) | 5 | (4 - 6) | 0.48 | (0.44 - 0.53) | |
| 200 | GGG 2 | 62 | (60 - 65) | 17 | (15 - 19) | 77 | (74 - 80) | 70 | (68 - 72) | 4 | (3 - 4) | 0.46 | (0.43 - 0.49) | |
| 200 | GGG 3 | 60 | (58 - 63) | 18 | (16 - 20) | 82 | (80 - 84) | 61 | (59 - 63) | 3 | (3 - 4) | 0.48 | (0.46 - 0.51) | |
| 200 | GGG 4 | 45 | (44 - 47) | 6 | (5 - 6) | 85 | (83 - 86) | 72 | (71 - 73) | 8 | (7 - 9) | 0.58 | (0.57 - 0.59) | |
| 200 | GGG 5 | 49 | (35 - 61) | 3 | (0.7 - 9) | 63 | (47 - 75) | 95 | (93 - 96) | 16 | (6 - 45) | 0.53 | (0.43 - 0.65) | |
| 300 | GGG 1 | 41 | (35 - 48) | 3 | (1 - 5) | 73 | (65 - 79) | 91 | (89 - 92) | 15 | (8 - 28) | 0.6 | (0.54 - 0.67) | |
| 300 | GGG 2 | 43 | (39 - 47) | 5 | (3 - 6) | 81 | (77 - 84) | 78 | (76 - 80) | 9 | (7 - 12) | 0.6 | (0.56 - 0.64) | |
| 300 | GGG 3 | 47 | (44 - 50) | 8 | (6 - 10) | 85 | (82 - 87) | 65 | (63 - 67) | 6 | (5 - 7) | 0.58 | (0.55 - 0.61) | |
| 300 | GGG 4 | 45 | (43 - 48) | 8 | (7 - 10) | 88 | (86 - 90) | 56 | (54 - 58) | 6 | (5 - 7) | 0.59 | (0.57 - 0.62) | |
| 300 | GGG 5 | 37 | (36 - 38) | 3 | (3 - 4) | 88 | (87 - 90) | 69 | (68 - 70) | 11 | (10 - 13) | 0.65 | (0.64 - 0.67) | |
| 400 | GGG 1 | 44 | (32 - 56) | 2 | (0.3 - 7) | 71 | (55 - 82) | 94 | (93 - 96) | 23 | (6 - 83) | 0.57 | (0.47 - 0.69) | |
| 400 | GGG 2 | 33 | (27 - 40) | 2 | (0.6 - 4) | 78 | (69 - 85) | 89 | (88 - 91) | 21 | (9 - 47) | 0.68 | (0.62 - 0.74) | |
| 400 | GGG 3 | 34 | (30 - 38) | 3 | (2 - 4) | 86 | (82 - 89) | 76 | (74 - 78) | 13 | (9 - 19) | 0.68 | (0.65 - 0.72) | |
| 400 | GGG 4 | 39 | (36 - 41) | 5 | (4 - 6) | 88 | (85 - 90) | 62 | (60 - 64) | 8 | (6 - 10) | 0.65 | (0.62 - 0.67) | |
| 400 | GGG 5 | 37 | (35 - 39) | 5 | (4 - 6) | 91 | (90 - 93) | 53 | (51 - 55) | 8 | (7 - 10) | 0.66 | (0.64 - 0.68) | |
| 500 | GGG 1 | 31 | (30 - 33) | 2 | (2 - 3) | 90 | (89 - 92) | 68 | (67 - 69) | 14 | (12 - 17) | 0.7 | (0.69 - 0.71) | |
| 500 | GGG 2 | 39 | (26 - 51) | 1 | (0.1 - 6) | 76 | (55 - 88) | 94 | (92 - 95) | 29 | (6 - 152) | 0.62 | (0.52 - 0.74) | |
| 500 | GGG 3 | 28 | (22 - 35) | 1 | (0.3 - 3) | 83 | (72 - 90) | 89 | (87 - 90) | 28 | (10 - 77) | 0.72 | (0.67 - 0.78) | |
| 500 | GGG 4 | 28 | (24 - 31) | 1 | (0.8 - 2) | 90 | (85 - 93) | 75 | (73 - 77) | 19 | (11 - 32) | 0.73 | (0.7 - 0.77) | |
| 500 | GGG 5 | 33 | (31 - 36) | 4 | (3 - 5) | 89 | (86 - 91) | 61 | (59 - 63) | 9 | (7 - 11) | 0.69 | (0.67 - 0.72) | |
| 1000 | GGG 1 | 32 | (30 - 34) | 3 | (2 - 4) | 93 | (92 - 95) | 51 | (50 - 53) | 11 | (8 - 13) | 0.7 | (0.69 - 0.72) | |
| 1000 | GGG 2 | 27 | (26 - 29) | 2 | (1 - 2) | 91 | (90 - 93) | 67 | (66 - 68) | 16 | (13 - 20) | 0.74 | (0.73 - 0.75) | |
| 1000 | GGG 3 | 34 | (22 - 46) | 1 | (0.1 - 6) | 75 | (54 - 87) | 93 | (91 - 95) | 28 | (5 - 152) | 0.67 | (0.57 - 0.79) | |
| 1000 | GGG 4 | 26 | (20 - 32) | 0.8 | (0.2 - 2) | 85 | (74 - 92) | 89 | (87 - 90) | 33 | (10 - 109) | 0.75 | (0.7 - 0.8) | |
| 1000 | GGG 5 | 24 | (21 - 27) | 1 | (0.5 - 2) | 91 | (86 - 94) | 74 | (72 - 76) | 21 | (12 - 39) | 0.77 | (0.74 - 0.8) | |
| **Abbreviation** PSA prostate-specific antigen; GGG Gleason Grade Group; CI confidence interval | | | | | | | | | | | | | |  |
