# Supplementary material for: Prediction of metastatic prostate cancer by prostate-specific antigen in combination with T stage and Gleason Grade: Nationwide, population-based register study
Source: PLoS One. 2020 Jan 29;15(1):e0228447. doi: 10.1371/journal.pone.0228447 (PMC6988964; doi:10.1371/journal.pone.0228447)
Supplement: Attachment — Submitted filename: METS_review answer.docx [file pone.0228447.s005.docx]

Dec 17 2019

Re: PONE-D-19-30700
Prediction of metastatic prostate cancer by prostate-specific antigen in combination with T stage and Gleason Grade. Nationwide, population-based register study
PLOS ONE

The image quality in Figure 1 needs to be improved.

*Answer: Figure 1 with higher resolution is included in the resubmission.*

Journal requirements:

When submitting your revision, we need you to address these additional requirements.

1. Please ensure that your manuscript meets PLOS ONE's style requirements, including those for file naming. The PLOS ONE style templates can be found at
[http://www.journals.plos.org/plosone/s/file?id=wjVg/PLOSOne_formatting_sample_main_body.pdf](http://www.journals.plos.org/plosone/s/file?id=wjVg/PLOSOne_formatting_sample_main_body.pdf) and [http://www.journals.plos.org/plosone/s/file?id=ba62/PLOSOne_formatting_sample_title_authors_affiliations.pdf](http://www.journals.plos.org/plosone/s/file?id=ba62/PLOSOne_formatting_sample_title_authors_affiliations.pdf)

*Answer: the ms has been updated accordingly*

2. We noticed you have some minor occurrence(s) of overlapping text with the following previous publication(s), which needs to be addressed:
[http://dx.doi.org/10.1016/j.eururo.2017.06.036](http://dx.doi.org/10.1016/j.eururo.2017.06.036)
[https://doi.org/10.1080/0284186X.2019.1662084](https://doi.org/10.1080/0284186X.2019.1662084)
[https://doi.org/10.1111/bju.14563](https://doi.org/10.1111/bju.14563)

In your revision ensure you cite all your sources (including your own works), and quote or rephrase any duplicated text outside the Methods section. Further consideration is dependent on these concerns being addressed.

*Answer: All studies use data from PCBaSe and there are therefore some parts of the methods section that is quite similar but where we reference other cohort profile publications. We reread all three papers and compared to the current study. We have not been able to find any overlapping text outside the methods section. If there are any sections that you deem necessary to reference please inform us of any overlapping text. Thank you.*

3. In the ethics statement in the manuscript and in the online submission form, please provide additional information about the patient records/samples used in your retrospective study. Specifically, please ensure that you have discussed whether all data/samples were fully anonymized before you accessed them and/or whether the IRB or ethics committee waived the requirement for informed consent. If patients provided informed written consent to have data/samples from their medical records used in research, please include this information.

*Answer:*

*Our statement regarding ethics in our submitted ms read: “This study was approved by the Research Ethics Board at Uppsala University and included all men diagnosed with prostate cancer in 2006-2016 in NPCR.*

*The Research Ethics Board at Uppsala University approved of the linkages in our project (PCBaSe 2016-239). We received a study file from Statistics Sweden and the National Board of Health and Welfare where the person identity number for men in the National prostate Cancer Register had been replaced by a code. This means that the data set is pseudononymized, but due to the large number of variables this dataset is still considered not anonymized when deleting this code. The following restrictions apply: we are not allowed to share data on individuals with other researchers, nor or we allowed upload such data on an open server. However, we can provide access to the dataset on a remote server on demand. On the Research platform, data can be uploaded and then accessed by external researchers. However, no individual data are allowed to leave the platform but aggregated data in the form of figures and Tables can be exported.*

*External researcher should contact the corresponding author who will direct the demand for data to the PCBaSe reference group who will then provider the access described above.*

4. We note that you have indicated that data from this study are available upon request. PLOS only allows data to be available upon request if there are legal or ethical restrictions on sharing data publicly. For information on unacceptable data access restrictions, please see [http://journals.plos.org/plosone/s/data-availability#loc-unacceptable-data-access-restrictions](http://journals.plos.org/plosone/s/data-availability#loc-unacceptable-data-access-restrictions).

In your revised cover letter, please address the following prompts:

a) If there are ethical or legal restrictions on sharing a de-identified data set, please explain them in detail (e.g., data contain potentially identifying or sensitive patient information) and who has imposed them (e.g., an ethics committee). Please also provide contact information for a data access committee, ethics committee, or other institutional body to which data requests may be sent.

*RE: See above*

b) If there are no restrictions, please upload the minimal anonymized data set necessary to replicate your study findings as either Supporting Information files or to a stable, public repository and provide us with the relevant URLs, DOIs, or accession numbers. Please see [http://www.bmj.com/content/340/bmj.c181.long](http://www.bmj.com/content/340/bmj.c181.long) for guidelines on how to de-identify and prepare clinical data for publication. For a list of acceptable repositories, please see [http://journals.plos.org/plosone/s/data-availability#loc-recommended-repositories](http://journals.plos.org/plosone/s/data-availability#loc-recommended-repositories).

We will update your Data Availability statement on your behalf to reflect the information you provide.

*RE: See above*

5. Please include captions for your Supporting Information files at the end of your manuscript, and update any in-text citations to match accordingly. Please see our Supporting Information guidelines for more information: [http://journals.plos.org/plosone/s/supporting-information](http://journals.plos.org/plosone/s/supporting-information).

*Answer: the ms has been updated accordingly*
